# Choline Regulates the Function of Bovine Immune Cells and Alters the mRNA Abundance of Enzymes and Receptors Involved in Its Metabolism *in vitro*

**DOI:** 10.3389/fimmu.2018.02448

**Published:** 2018-10-25

**Authors:** Miriam Garcia, Laman K. Mamedova, Barbara Barton, Barry J. Bradford

**Affiliations:** ^1^Department of Animal Sciences and Industry, Kansas State University, Manhattan, KS, United States; ^2^Balchem Corporation, New Hampton, NY, United States

**Keywords:** choline, transition dairy cow, immune cells, phagocytosis, proliferation, mRNA abundance

## Abstract

Dietary choline can impact systemic immunity, but it remains unclear whether this is primarily via direct impacts on immune cells or secondary effects of altered metabolic function. To determine whether increased choline concentrations (3.2, 8.2, 13.2 μM) in cell culture alter the function of bovine innate and adaptive immune cells, we isolated cells from dairy cows in early and mid-lactation as models of immuno-compromised and competent cells, respectively. Phagocytic and killing capacity of isolated neutrophils were linearly diminished with increasing doses of choline. In contrast, lymphocyte proliferation was linearly enhanced with increasing doses of choline. Furthermore, increasing doses of choline increased the mRNA abundance of genes involved in the synthesis of choline products (betaine, phosphatidylcholine, and acetylcholine) as well as muscarinic and nicotinic acetylcholine receptors in a quadratic and linear fashion for neutrophils and monocytes, respectively. Phagocytic and killing capacity of neutrophils and proliferation of lymphocytes were not affected by stage of lactation or its interaction with choline or LPS. In neutrophils from early lactation cows, choline linearly increased the mRNA abundance of muscarinic and nicotinic cholinergic receptors, whereas choline-supplemented monocytes from mid-lactation cows linearly increased the mRNA abundance of several genes coding for choline metabolism enzymes. These data demonstrate that choline regulates the inflammatory response of immune cells and suggest that the mechanism may involve one or more of its metabolic products.

## Introduction

Choline is a quaternary amine obtained from the diet which can also be synthesized by several organs, primarily the liver (1). Choline is a versatile compound, serving as an essential precursor of several different products. One, betaine, is primarily synthesized in the liver and kidney and serves as a methyl donor, aiding in the regeneration of methionine and S-adenosylmethionine (2). Phosphatidylcholine is the major component of cellular membranes, and in the rat liver it enhances the synthesis and export of very low-density lipoprotein, reducing hepatic fat accumulation (3). Lastly, the neurotransmitter acetylcholine is primarily synthesized in cholinergic neurons, where activated muscarinic and nicotinic receptors trigger second messengers (4), but it has also been detected in bovine blood at ~40× greater concentrations than in human blood (5). More recently, some studies have identified muscarinic and nicotinic cholinergic receptors in innate and adaptive immune cells from rodents and humans (6–8).

Dairy cows in early lactation have dysregulated immune function, which impacts neutrophils as well as circulating monocytes and lymphocytes (9, 10). This immune dysfunction is likely driven, at least in part, by incomplete metabolic adaptations to the extreme increase in demand for nutrients to cope with the onset of lactation (11, 12). Decreasing initial milk yield and nutrient demand through prolactin blockade increased oxidative burst functionality in neutrophils, although it also decreased lymphocyte proliferation (13). Supplementation of rumen-protected choline (RPC) to periparturient cows improved their performance and reduced the incidence of diseases (14–17). Furthermore, monocytes isolated from periparturient cows fed RPC demonstrated an enhanced killing capacity (18). Nevertheless, in rodents and human cell lines, supplementation with the choline metabolites betaine and phosphatidylcholine during colitis-associated cancer and *C. difficile* toxin A infestation, respectively, had anti-inflammatory effects resulting in reduced tumor growth and improved cell integrity (19, 20). Therefore, the direct effects of choline on immunity vs. those mediated by impacts on metabolic function or secondary product formation may differ.

Despite the productivity and health benefits of supplementing RPC to dairy cows, the circulating concentration of choline ions is fairly stable. Zenobi et al. (17) supplemented transition cows with increased levels of RPC to provide, over basal diet concentrations, 0, 6.5, 12.9, 19.4, and 25.8 g/d of choline ions, and reported no change in plasma choline ion concentrations, which averaged 4.3 μM. Nevertheless, the authors reported trends for a linear increase in concentrations of lysophosphatidylcholine and sphingomyelin with increasing dose of RPC. Furthermore, Artegoitia et al. (21) reported that plasma concentration of choline ions is stable across the entire lactation, but that the total concentration of choline metabolites changes dramatically. Total choline metabolites in plasma were 5× and 13× greater in mid and late lactation, respectively, relative to early lactation. A recent study reported concentrations of plasma choline ions of 7.6 and 13.1 μM in response to an abomasal infusion of 12.5 and 25 g choline ions per day, respectively (22). De Veth et al. (22) suggested that the lipid coating used to prevent ruminal degradation of choline chloride may diminish its bioavailability. Studies evaluating the functional impacts and potential mechanisms of action of choline in dairy cow immune cells at different stages of lactation are lacking. We hypothesized that immune cells regulate their function in response to varying choline supply, and that cows in early lactation, who have lower concentrations of choline metabolites compared with mid- or late lactation dairy cows, would benefit from supraphysiological concentrations of choline.

Isolation and *in vitro* incubation of immune cells are well-established techniques appropriate to understand signaling mechanisms while avoiding multiple confounding effects of nutrients on systemic physiology. Our first objective was to determine whether choline treatment *in vitro* has a direct impact on the inflammatory response of bovine immune cells. After finding that increasing doses of choline enhanced lymphocyte proliferation but had anti-inflammatory effects on neutrophils and monocytes, our second objective was to assess choline effects on mRNA abundance of genes involved in choline metabolism and inflammatory responses of these immune cells.

## Materials and methods

All procedures involving the use of live animals were approved and carried out in accordance with the recommendations set forth by the Institutional Animal Care and Use Committee at Kansas State University.

### Animals

Twenty multiparous Holstein cows in early (*n* = 10, 6.9 ± 1.8 days in milk) and mid (*n* = 10, 123 ± 3.4 days in milk) lactation were used for immune cell collection. Cows were without clinical signs of disease (including visually normal milk), displaying normal body temperature (38.7 ± 0.34°C, mean ± SD, range: 38.3–39.5°C), and somatic cell counts (early lactation range: 13–264 × 10^3^ cell/mL; mid-lactation range: 13–62 × 10^3^ cell/mL). All cows were housed and fed in free stalls, had free access to water, and were milked thrice daily. Cows were fed *ad libitum* a lactation diet formulated to meet all nutritional requirements (23). The diet provided 65.5 and 3,119 g/d of metabolizable methionine and protein, respectively, at 25.9 kg/d of dry matter intake, as estimated by the Cornell Net Carbohydrate and Protein System version 6.55 (NDS Professional, RUM&N Sas, Emilia, Italy). On a given day, a balanced number of cows per stage of lactation were bled (150 mL) via jugular venipuncture. Blood samples were immediately transferred in crushed ice to the laboratory and processed within 30 min.

### Cell isolation

**Figure 1:**
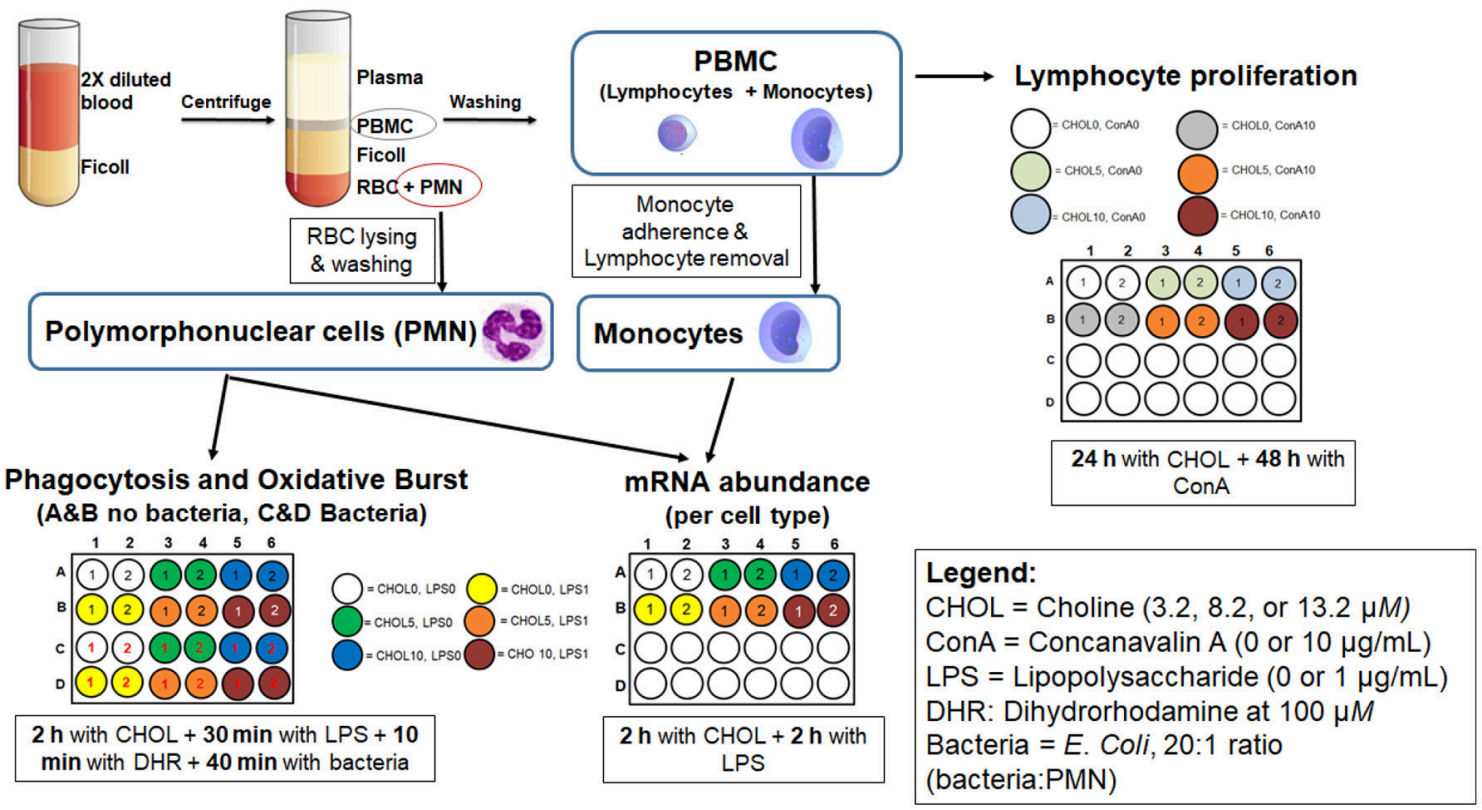
Schematic representation of the methods used for cell isolation and culture. All assays were run in isolated cells from 20 cows (10 early- and 10 mid-lactation cows), were performed in duplicate wells (technical replicate) and individually analyzed for phagocytosis, oxidative burst, and proliferation. For mRNA abundance and TNF-α in spent media, cells, and media from two cultured wells were pooled.

The cell isolation procedure was performed as described elsewhere (24, 25) with minor modifications for isolation of different cell types. Briefly, diluted blood (2× with 2 mM EDTA in phosphate-buffered saline) underwent a differential gradient centrifugation step using Ficoll-paque PLUS (GE Healthcare, Pittsburgh, PA). After removal of the diluted plasma, the opaque phase containing peripheral blood mononuclear cells (PBMC, Figure 1) was collected in a separate tube. These cells underwent a series of washing steps to remove platelets and retain primarily monocytes and lymphocytes. The bottom phase was subjected to RBC lysis to retain polymorphonuclear cells (PMN) only. Cell viability by trypan blue exclusion was measured after PBMC (96.5 ± 2.8%, mean ± SD) and PMN (99.2 ± 1.0%, mean ± SD) isolation.

To confirm the purity of the PMN fraction, cells were microscopically counted on their corresponding smears stained with modified Wright-Giemsa stain (Protocol-Hema3, Biochemical Sciences, Swedesboro, NJ), revealing 97.7 ± 2.6% PMN. Furthermore, we have previously reported that the proportion of neutrophils relative to the pool of neutrophils, eosinophils, and basophils from the blood of dairy cows is about 92% (26). Given the high proportions of neutrophils in the isolated PMN fraction, we will refer to this fraction as neutrophils instead of PMN.

Monocytes were microscopically counted on smears from the PBMC fraction stained with modified Wright-Giemsa stain, revealing 12.7 ± 1.4% monocytes in PBMC. Monocytes were further isolated from PBMC by adherence. Briefly, PBMC were diluted at 1 × 10^7^ cells/mL with medium 199 (ThermoFisher Scientific, Waltham, MA) and incubated at 37°C and 5% CO_2_ for 2 h. After incubation, the medium containing lymphocytes was removed, and the plate was washed to remove any remaining lymphocytes.

### Treatments and cell culture

A simplified outline of treatments and cell culture is presented in Figure 1. The doses of choline treatment were selected based on basal concentrations (4 μM) of free choline in plasma of cows during the first week of lactation (21) and plasma concentrations achievable with abomasal choline infusion (22). Choline chloride (Sigma Aldrich Inc.) was used as a source of supplemental choline. The commercial media used was selected for its relatively low concentration of choline chloride (0.5 mg/L; Media-199, Gibco, ThermoFisher Scientific). The basal media contained 10% FBS (Gibco™, ThermoFisher Scientific), for which choline concentration was not available. Considering only the contribution of M-199 media, the concentration of choline ion in the basal media was 3.2 μM. The choline treatments provided an additional 5 or 10 μM choline. Therefore, the final concentrations of choline, accounting for the choline present in the basal media, were 3.2 (**CHO3**), 8.2 (**CHO8**), and 13.2 (**CHOL13**) μM. Because choline chloride was used to provide the supplemental choline in CHO8 and CHO13, these treatments also added 5 and 10 μM additional chloride. However, the additional chloride is a tiny fraction of the 125 mM concentration in M-199 media or the 107 mM concentration in bovine plasma (27), suggesting that the additional chloride was not impactful.

#### Lymphocyte proliferation

PBMC from early (*n* = 10) and mid-lactation (*n* = 10) cows were resuspended (5 × 10^6^ cells/mL final concentration, 100 μL/well final volume) with medium 199 containing 10% FBS and 2% of 10,000 units of penicillin and 10,000 μg of streptomycin per microliter (Gibco, ThermoFisher). Cells were incubated for 24 h with one of the three choline treatments followed by a 48 h incubation with or without Con A (10 μg/mL final concentration, Sigma-Aldrich Co.). After incubation, 10 μL of BrdU reagent (Abcam, Cambridge, MA) was added to each well and incubated for 24 h to allow incorporation of BrdU in proliferating cells.

#### Phagocytosis and oxidative burst assays

Neutrophils (3 × 10^6^ cells/mL final concentration, 1 mL/well final volume) from cows in early (*n* = 9) and mid-lactation (*n* = 9) were resuspended in media 199 containing 10% FBS, assigned one of three doses of choline, and cultured for 2 h at 37°C and 5% CO_2_. Due to methodological errors, samples from two cows were excluded from this analysis. Next, cells were primed with or without LPS (1 μg/mL, final concentration, *Escherichia coli* O55:B5, Sigma-Aldrich Co.) for 30 min followed by the addition of dihydrorhodamine (100 μM, final concentration, Sigma-Aldrich). Ten minutes after incubation with dihydrorhodamine, cells were challenged with *E. coli* covalently labeled with Texas Red® (Molecular Probes Inc.) at a ratio of 20:1 (bacteria:neutrophil) and returned to incubation for 40 min. After incubation, neutrophil activity was stopped by cold shock. Lastly, the cells were washed and pelleted three times and finally reconstituted in 200 μL of phosphate-buffered saline for fluorescence measurement.

#### mRNA abundance

Neutrophils (5 × 10^6^/mL, final concentration, 1 mL/well final volume) and monocytes (~1.2 × 10^6^/mL, based on % of monocytes in PBMC, final concentration, 1 mL/well final volume) from cows in early (*n* = 10) and cows in mid-lactation (*n* = 10) were resuspended in media 199 containing 10% FBS (Gibco, ThermoFisher Scientific), assigned one of three doses of choline, and cultured for 2 h at 37°C and 5% CO_2_. After incubation, choline-treated cells were challenged with or without LPS (1 μg/mL, final concentration) and cultured at 37°C and 5% CO_2_ for another 2 h. After final incubation, spent media was collected, the cell pellet was lysed with RNA isolation lysing solution (PureLink RNA Mini Kit, ThermoFisher Scientific), and both were stored at −80°C for further analysis of TNF-α and mRNA abundance, respectively.

### Assays

#### Proliferation of PBMC

After concluding the incubation with BrdU, the cells were fixed to denature the DNA and enable antibody binding. A commercial kit provided all the reagents needed to fix cells and to perform an ELISA assay to measure cell proliferation (BrdU Cell Proliferation ELISA kit, Abcam, Cambridge, MA). The BrdU absorbance value for each treatment was divided by the absorbance value obtained for CHOL3 cells without Con A to quantify relative cell proliferation rates.

#### Flow cytometry

Neutrophil activity was assessed using a capillary flow cytometer (Guava EasyCyte Mini Flow Cytometry System; Millipore Sigma, Billerica, MA) and the data were analyzed using the Guava ExpressPro option within CytoSoft 5.3 software. Five thousand cells were counted per sample. Neutrophils were gated based on side scatter- and forward scatter-area. Cells incubated without bacteria but with dihydrorhodamine were negative controls and were used to correct for nonspecific fluorescence. Green and red voltages and fluorescence compensation settings were optimized before samples acquisition. The percentage of neutrophils that contained red fluorescence represents the phagocytosis of Texas red-labeled *E. coli*, and the percentage of neutrophils with green fluorescence represents oxidative burst. Additionally, the mean fluorescence intensity of the red and green wavelengths is a proxy for the mean number of bacteria phagocytized by neutrophils and the mean amount of reactive oxygen species (ROS) produced per neutrophil, respectively.

#### Tumor necrosis factor-α

The spent media collected after cell incubation was analyzed for concentrations of TNF-α using a bovine TNF-α commercial kit (VetSet Elisa Development Kit; Kingfisher Biotech Inc., St. Paul, MN) following the manufacturer's protocol with some modifications. Standards were prepared with the basal media used to resuspend cells (M-199 and 10% FBS). Samples were run in duplicate. The minimum consistently detectable concentration of TNF-α was 8 pg/mL, and samples that were undetectable were assigned a 0 concentration. The intra- and inter-assay coefficients of variation were 13.2 and 13.8%, respectively.

#### mRNA abundance

Cell lysates were homogenized, and RNA was extracted according to manufacturer recommendations (PureLink RNA Mini Kit, ThermoFisher Scientific). RNA samples from two cows in each stage of lactation having the lowest RNA concentration, for each cell type, were not included in further analyses. A subset of RNA samples from each treatment was analyzed for RNA integrity using an Agilent 2100 Bioanalyzer (Agilent Technologies, Santa Clara, CA), resulting in an average RNA integrity number of 7.2 for each cell type. For quantitative real-time PCR (qPCR), a fixed amount of RNA (340 and 180 μg for monocytes and neutrophils, respectively) was reverse transcribed using iScript Reverse Transcription Supermix (Bio-Rad Laboratories Inc., Hercules, CA). Quantitative PCR was performed using iTaq Universal SYBR Green Supermix (Bio-Rad Laboratories Inc.) with Invitrogen custom primers (ThermoFisher Scientific). The PCR reactions used cDNA derived from 7.8 and 4.1 ng of RNA from monocytes and neutrophils, respectively. Primers and reaction efficiency values are shown in Supplemental Table 1. The threshold cycle (Ct)-values from target genes were normalized to the geometric mean of *RPS9* and *RPS15* Ct-values, which were not influenced by treatments, and were selected as the most stable ones among four evaluated genes (along with *GAPDH* and *ACTB*) using the Excel-based tool BestKeeper (28). The ΔCt-values were calculated, linearized using the formula 2^−ΔCt^ (29), and are presented as arbitrary units (AU) on this scale.

### Statistical analyses

The MIXED procedure of SAS (SAS/STAT version 9.4; SAS Institute Inc., Cary, NC) was used for statistical analysis. The design was a randomized block (stage of lactation) with a factorial arrangement of treatments that varied according to the assay. All data, except proliferation that was log-transformed and mRNA abundance (ΔCt) that was not transformed, were transformed to the square root before statistical analysis to attain a normal residual distribution during statistical analysis. For phagocytosis, oxidative burst, mRNA abundance, and concentrations of TNF-α in media, the data were analyzed using a model that included the fixed effects of stage of lactation, choline, LPS, their interaction, and the random effect of cow. The analyzed contrasts were: (1) CHOL3 vs. supplemental choline, (2) CHOL8 vs. CHOL13, (3) CHOL3 vs. supplemental choline × LPS, (4) CHOL8 vs. CHOL13 × LPS, (5) CHOL3 vs. supplemental choline × stage of lactation, and (6) CHOL8 vs. CHOL13 × stage of lactation. For cell proliferation, the model included stage of lactation, choline, Con A, and their interactions. For both models, the denominator degrees of freedom were estimated with the Kenward-Roger specification in the model statement except for a few variables for which the Satterthwaite option was chosen due to unequal variances. The data are presented as back-transformed LSM ± back-transformed standard error of the mean (SEM). For back-transformation of the SEM of log-transformed data, the back-transformed upper and lower limit of the confidence interval was calculated for each mean, and the pooled mean confidence interval (on the back-transformed scale) was determined as the SEM (30). The back-transformed SEM of data analyzed with a square-root transformation was estimated as 2 × LSM × SEM [both on transformed scale; (31)]. Statistical differences were declared significant if *P* ≤ 0.05.

## Results

### LPS-mediated inflammation

The LPS dose used in this study induced an inflammatory response in the cells examined. Indeed, LPS challenge increased the mRNA abundance of *TLR4, NFKB1, TNFA*, and *ELANE* in neutrophils (*P* ≤ 0.01, Table 1) and that of *NFKB1* and *TNFA* in monocytes (*P* ≤ 0.001, Table 2). In contrast, LPS suppressed *CASP7* mRNA abundance in neutrophils (Table 1) and *TLR4* mRNA in monocytes, particularly those from early lactation cows (Table 2).

**Table 1:** Impact of stage of lactation and LPS on mRNA abundance (2^−ΔCt^ × 10^−3^AU) of genes involved in choline metabolism and inflammatory response in neutrophils from lactating dairy cows *in vitro*^a^^,^^b^.

	**Stage of lactation (SL)**				**SEM^c^**	***P*****-value**		
	**Early**		**Mid**			
	**LPS0**	**LPS1**	**LPS0**	**LPS1**		**SL**	**LPS**	**SL × LPS**
**CHOLINE METABOLISM**								
*SLC5A7*	0.86	1.02	0.86	0.90	0.38	0.92	0.45	0.68
*CHDH*	1.18	0.99	0.77	1.01	0.33	0.65	0.68	0.06
*CHKA*	212	296	197	279	52	0.76	0.12	0.97
*ACHE*	2.20	2.02	2.61	2.73	0.60	0.50	0.78	0.38
*CHRM5*	0.68	0.65	0.76	0.87	0.23	0.64	0.71	0.41
*CHRNA7*	1.06	1.19	0.94	0.88	0.50	0.75	0.87	0.58
**INFLAMMATORY RESPONSE**								
*TLR4*	801	1127	1645	2470	218	<0.001	0.01	0.81
*NFKB1*	567	1508	914	1769	162	0.02	<0.001	0.23
*TNFA*	75	1948	140	1320	322	0.71	<0.001	0.12
*ELANE*	52.2	85.9	81.3	113.7	28.3	0.46	<0.001	0.005
*H2A*	174	194	232	246	32	0.23	0.10	0.65
*CASP3*	35.7	27.0	36.7	36.5	7.0	0.55	0.18	0.19
*CASP7*	88.6	74.5	117.1	91.7	18.1	0.38	<0.001	0.54

a *Mean values per treatment correspond to cells from 18 cows (9 in early and 9 in mid-lactation) that were or not challenged with LPS (1 μg/mL)*.

b *Cells were cultured with either 3.2 (CHOL3), 8.1 (CHOL8), or 13.2 (CHOL13) μM choline*.

c *Pooled standard error of the mean*.

**Table 2:** Impact of stage of lactation and LPS on mRNA abundance (2^−ΔCt^ × 10^−3^AU) of genes involved in choline metabolism and inflammatory response in monocytes from lactating dairy cows *in vitro*^a^^,^^b^.

	**Stage of lactation (SL)**				**SEM^c^**	***P*****-value**		
	**Early**		**Mid**			
	**LPS0**	**LPS1**	**LPS0**	**LPS1**		**SL**	**LPS**	**SL × LPS**
**CHOLINE METABOLISM**								
*SLC5A7*	0.68	0.51	0.51	0.67	0.12	0.98	0.99	0.009
*CHDH*	0.65	0.66	0.62	0.74	0.10	0.88	0.28	0.35
*CHKA*	19.9	16.9	21.0	20.3	3.5	0.51	0.58	0.72
*ACHE*	1.89	1.70	1.93	2.23	0.42	0.62	0.45	<0.001
*CHAT*	0.25	0.24	0.18	0.26	0.07	0.74	0.20	0.20
*CHRM1*	0.12	0.12	0.08	0.13	0.02	0.57	0.11	0.06
*CHRM5*	0.24	0.21	0.17	0.27	0.04	0.84	0.17	0.01
*CHRNA7*	0.41	0.36	0.27	0.47	0.10	0.83	0.18	0.03
**INFLAMMATORY RESPONSE**								
*TLR4*	87.0	65.3	66.6	62.0	22.0	0.71	<0.001	0.04
*NFKB1*	257	349	240	366	44	0.97	<0.001	0.22
*TNFA*	71	344	79	264	54	0.76	<0.001	0.48
*H2A*	161.6	183.4	152.4	191.1	21.7	0.95	0.17	0.70
*CASP3*	6.78	6.60	5.91	6.80	1.00	0.80	0.24	0.08
*CASP7*	6.92	6.57	8.37	8.47	1.38	0.23	0.91	0.86

a *Mean values per treatment correspond to cells from 18 cows (9 in early and 9 in mid-lactation) that were or not challenged with LPS (1 μg/mL)*.

b *Cells were cultured with either 3.2 (CHOL3), 8.1 (CHOL8), or 13.2 (CHOL13) μM choline*.

c *Pooled standard error of the mean*.

The release of TNF-α in the spent media from neutrophils was not impacted by LPS challenge (15.9 vs. 23.4 ± 6.8 pg/mL without and with LPS, respectively, *P* = 0.28). In contrast, LPS strongly increased the production of TNF-α by monocytes (1,135 vs. 70 ± 169 pg/mL, *P* < 0.001).

### Innate and adaptive immune cells have opposite functional responses to increasing doses of choline

**Figure 2:**
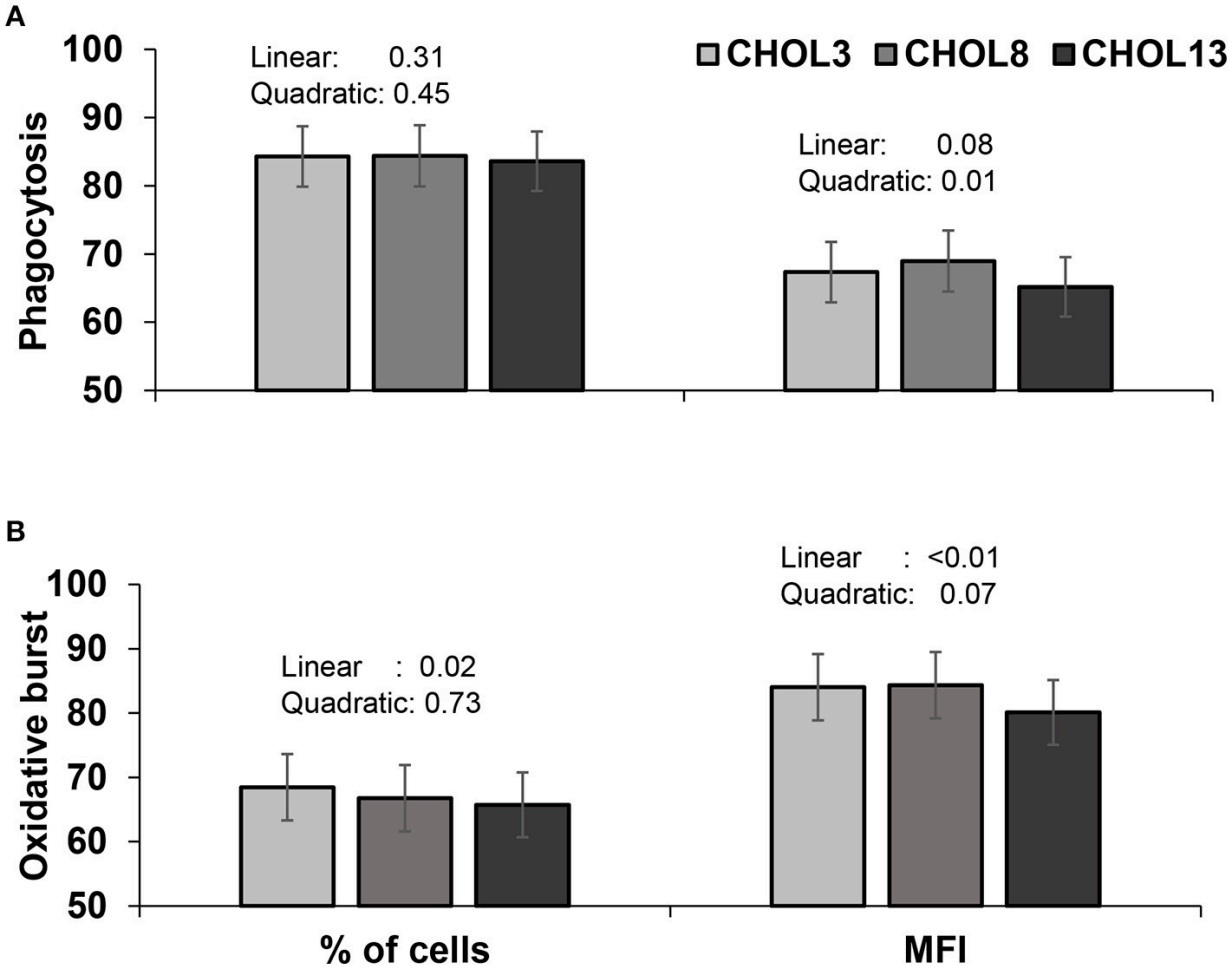
Phagocytosis **(A)** and oxidative burst **(B)** activities of neutrophils isolated from blood of 18 cows (9 in early- and 9 in mid-lactation). Cells were cultured in two wells per sample and were assayed individually. Cells were incubated with the corresponding choline treatment (3.2, 8.2, or 13.2 μM) in duplicate wells and assayed individually. All cells were then incubated with dihydrorhodamine (100 μM) for 10 min followed by the addition of labeled *E. coli* (20:1 ratio of bacteria:neutrophil). Percentage of cells carrying out phagocytosis and oxidative burst and mean fluorescence intensity (MFI) of each were determined by flow cytometry. Data depicted represent the back-transformed (square root) LSM and SEM. Linear and quadratic *P*-values refer to the effect of choline doses.

**Figure 3:**
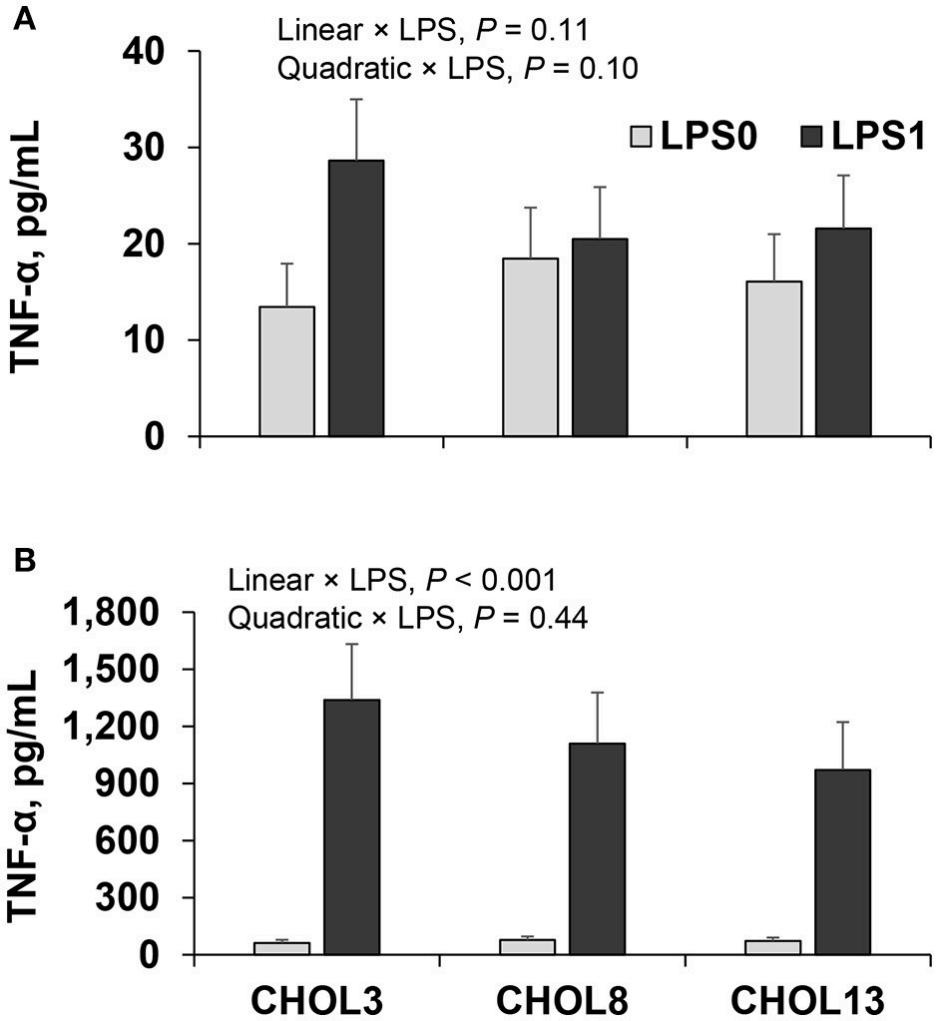
TNF-α in spent media of neutrophils **(A)** and monocytes **(B)** isolated from 18 cows (9 in early- and 9 in mid-lactation), cells were cultured in two wells per sample, but media from those wells were pooled before assayed for TNF-α. Cells were incubated with the corresponding choline treatment (3.2, 8.2, or 13.2 μM) for 2 h followed by a challenge] with LPS (0 or 1 μg/mL) for 2 h. Data depicted represent the back-transformed square root of LSM and SEM. Linear and quadratic *P*-values refer to the effect of choline doses.

Our first objective was to evaluate whether choline supplementation has an effect on immune cells in the absence of systemic metabolic effects. For neutrophils, the percentage of cells demonstrating phagocytic activity was not altered by choline (Figure 2A), but the proportion of cells exhibiting an oxidative burst decreased linearly as choline supplementation increased (*P* < 0.05, Figure 2B). The average number of *E. coli* phagocytized and the mean ROS produced per neutrophil were decreased to a greater extent with the highest dose of choline used, in a quadratic and linear manner for the former and latter, respectively (*P* ≤ 0.01, Figures 2A,B). The mRNA abundance of inflammatory genes was scarcely affected by choline supplementation (Table 3). A greater dose of choline was needed to reduce the abundance of *TLR4* (quadratic effect*, P* = 0.02) coding for the LPS pattern recognition receptor. The mRNA abundance of the transcription factor NF-κB (*NFKB1*) and *TNFA* were not altered with choline supplementation. Nevertheless, concentrations of TNF-α tended to decrease with the highest dose of choline, for the LPS-challenged neutrophils only (quadratic × LPS, *P* = 0.10, Figure 3A).

**Table 3:** mRNA abundance (2^−ΔCt^ × 10^−3^ AU) of genes involved in choline metabolism and inflammatory response in neutrophils from lactating dairy cows supplemented with choline *in vitro*^a^.

	**Treatment**^**b**^			**SEM^c^**	***P*****-value**	
	**CHOL3**	**CHOL8**	**CHOL13**		**Linear**	**Quadratic**
**CHOLINE METABOLISM**						
*SLC5A7*	0.74	1.22	0.83	0.28	0.47	<0.01
*CHDH*	0.79	1.16	1.02	0.24	0.10	0.05
*CHKA*	228	251	249	26	0.01	0.08
*ACHE*	2.18	2.49	2.46	0.43	0.17	0.34
*CHRM5*	0.65	0.87	0.70	0.17	0.63	0.04
*CHRNA7*	0.75	1.53	0.89	0.38	0.43	0.001
**INFLAMMATORY RESPONSE**						
*TLR4*	1,397	1,430	1,331	102	0.03	0.02
*NFKB1*	1,079	1,082	1,092	71	0.57	0.87
*TNFA*	416	390	410	67	0.80	0.23
*ELANE*	82	79	80	19	0.61	0.47
*H2A*	212	198	219	23	0.59	0.14
*CASP3*	36.9	30.5	33.9	5.2	0.52	0.18
*CASP7*	92.5	91.6	91.2	12.9	0.84	0.96

a *Mean values per treatment correspond to cells from 16 cows (8 in early and 8 in mid-lactation)*.

b *Cells were cultured with either 3.2 (CHOL3), 8.1 (CHOL8), or 13.2 (CHOL13) μM choline*.

c *Pooled standard error of the mean*.

For monocytes, the abundance of *TLR4* decreased linearly as choline concentration increased (*P* < 0.001, Table 4), but the decrease tended to be more dramatic in LPS-challenged monocytes (81, 76, 72, and 73, 71, 50 ± 6 × 10^−3^ AU for CHOL3, CHOL8, CHO13 in LPS0 and LPS1, respectively; *P* = 0.04). As expected, a linear decrease in abundance of *TLR4* was coupled with a linear decrease in abundance of *NFKB1*, responsible for inducing the synthesis of pro-inflammatory cytokines, also, similar to that of *TLR4*, the impact of choline was more drastic in LPS-challenged monocytes (258, 253, 235, and 400, 379, 301 ± 25 × 10^−3^ AU for CHOL3, CHOL8, CHO13 in LPS0 and LPS1, respectively; *P* = 0.10). Although the abundance of *TNFA* was not altered by choline, the release of TNF-α in the media of LPS-challenged monocytes decreased linearly as choline supplementation increased (Figure 3B, *P* < 0.05). The mRNA abundance of *H2A*, coding for histone H2A, was quadratically increased with increasing dose of choline (*P* = 0.03).

**Table 4:** mRNA abundance (2^−ΔCt^ × 10^−3^AU) of genes involved in choline metabolism and inflammatory response in monocytes from lactating dairy cows supplemented with choline *in vitro*^a^.

	**Treatment**^**b**^			**SEM^c^**	***P*****-value**	
	**CHOL3**	**CHOL8**	**CHOL13**		**Linear**	**Quadratic**
**CHOLINE METABOLISM**						
*SLC5A7*	0.41	0.59	0.84	0.09	<0.001	0.97
*CHDH*	0.53	0.68	0.83	0.08	<0.001	0.80
*CHKA*	20.0	20.7	17.8	1.81	0.01	0.02
*ACHE*	1.93	1.90	1.94	0.29	0.93	0.60
*CHAT*	0.14	0.25	0.36	0.06	<0.001	0.50
*CHRM1*	0.07	0.11	0.16	0.02	<0.001	0.70
*CHRM5*	0.15	0.23	0.30	0.03	<0.001	0.44
*CHRNA7*	0.21	0.37	0.62	0.08	<0.001	0.94
**INFLAMMATORY RESPONSE**						
*TLR4*	76.9	73.3	59.8	15.5	<0.001	0.16
*NFKB1*	321	310	266	31	<0.001	0.26
*TNFA*	142	158	146	21	0.74	0.13
*H2A*	169	182	164	12	0.59	0.03
*CASP3*	6.61	6.79	6.16	0.72	0.23	0.23
*CASP7*	7.51	7.83	7.27	0.73	0.58	0.24

a *Mean values per treatment correspond to cells from 16 cows (8 in early and 8 in mid-lactation)*.

b *Cells were cultured with either 3.2 (CHOL3), 8.1 (CHOL8), or 13.2 (CHOL13) μM choline*.

c *Pooled standard error of the mean*.

**Figure 4:**
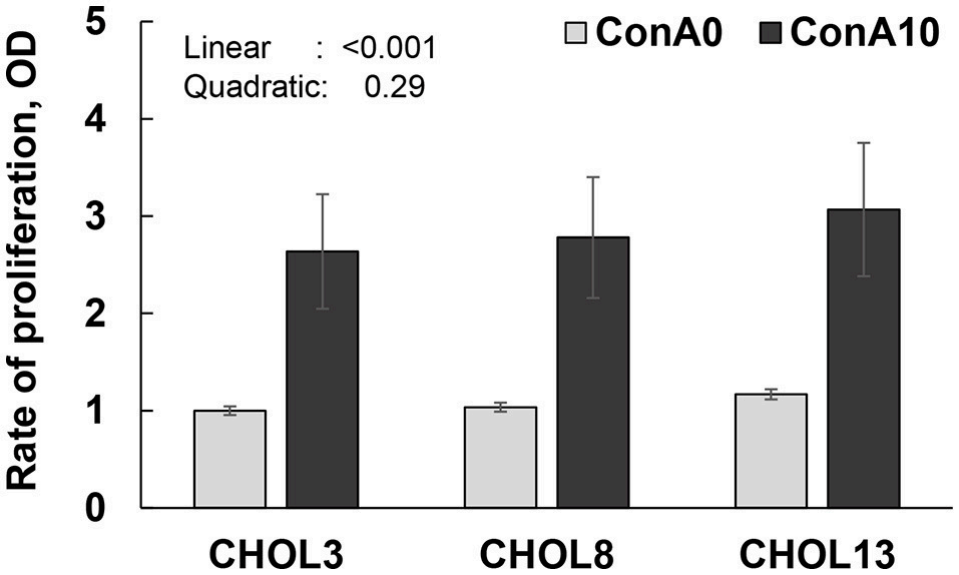
Proliferation rate (OD: optical density; values in reference to CHOL3 without Con A) of lymphocytes from isolated PBMC from 18 cows (9 in early- and 9 in mid-lactation). Cells were cultured in two wells per sample and were assayed individually. Cells were incubated with the corresponding choline treatment (3.2, 8.2, or 13.2 μM). For 24 h followed by 48 h incubation with Con A (0 or 10 μg/mL). Cells were then incubated with BrdU for 24 h to allow its incorporation into proliferating DNA. Data depicted represent the back-transformed square root of LSM and SEM. Linear and quadratic refer to the effect of choline doses. No interaction of linear or quadratic effect of choline with Con A was observed (*P* > 0.70).

Lymphocyte proliferation increased linearly (*P* < 0.001) as choline supplementation increased. Although proliferation was greatly enhanced by Con A, there was no evidence of a choline × Con A interaction (*P* > 0.70), suggesting that their effects were additive rather than synergistic (Figure 4).

### mRNA abundance of enzymes and receptors for choline metabolism products are increased with increasing dose of choline

The mRNA abundance of the choline receptor *SLC5A7* increased quadratically in neutrophils (*P* < 0.01, Table 3) and linearly in monocytes (*P* < 0.001, Table 4), with greatest abundance observed at the CHOL8 and CHOL13 dose for neutrophils and monocytes, respectively. LPS challenge altered the impact of choline; the quadratic effect of choline on the expression of *SLC5A7* tended to be stronger in unchallenged neutrophils (0.57, 1.39, 0.82 and 0.96, 1.07, 0.85 ± 0.23 × 10^−3^ AU for CHOL3, CHOL8, CHO13 in LPS0 and LPS1, respectively; *P* = 0.07). For monocytes, the linear effect of choline on *SLC5A7* tended to be greater in LPS-challenged monocytes (0.47, 0.60, 0.72, and 0.35, 0.58, 0.99 ± 0.11 × 10^−3^ AU for CHOL3, CHOL8, CHO13 in LPS0 and LPS1, respectively; *P* = 0.07).

The mRNA of choline dehydrogenase (*CHDH*), responsible for the synthesis of betaine aldehyde, increased in a quadratic manner in neutrophils (*P* = 0.05, Table 3), with greatest abundance observed with CHOL8. For monocytes, *CHDH* abundance increased linearly as choline supplementation increased (*P* < 0.001, Table 4). The mRNA of choline kinase alpha (*CHKA*), the enzyme responsible for the first reaction in the synthesis of phosphatidylcholine, increased linearly as choline supplementation increased (*P* ≤ 0.01), although the greater increase was or tended to be observed in CHOL8 cells (quadratic, *P* = 0.08 and 0.02, for neutrophils and monocytes, respectively). Challenge of neutrophils with LPS did not impact the effect of choline on *CHKA* abundance, but for monocytes, LPS challenge linearly decreased the abundance of *CHKA* (20.0, 21.4, 20.0, and 20, 20, 15.8 ± 2.7 × 10^−3^ AU for CHOL3, CHOL8, CHO13 in LPS0 and LPS1, respectively; *P* = 0.01).

The mRNA abundance of choline acyltransferase (*CHAT*), the enzyme responsible for the synthesis of acetylcholine, was undetectable in pooled cDNA from neutrophil samples; hence it was not further analyzed (descriptive Ct data for all targets are provided in Supplemental Table 2). In monocytes, *CHAT* abundance increased linearly as choline supplementation increased (*P* < 0.001, Table 4). Furthermore, the mRNA abundance of acetylcholinesterase (*ACHE*), the enzyme responsible for the breakdown of acetylcholine, was not affected by choline supplementation or its interaction with LPS in neutrophils or monocytes.

Although *CHAT* was undetected in neutrophils, the abundance of the nicotinic acetylcholine receptor (*CHRNA7*) and that of the muscarinic acetylcholine receptor (*CHRM5*) were increased quadratically with choline supplementation, with the greatest abundance observed in CHOL8 (*P* < 0.05, Table 3). However, for LPS-challenged neutrophils, a tendency for a linear decrease in *CHRM5* with increasing choline was observed (0.54, 0.96, 0.72, and 0.80, 0.78, 0.67 ± 0.14 × 10^−3^ AU, for CHOL3, CHOL8, CHO13 in LPS0 and LPS1, respectively, *P* = 0.09). In monocytes, the mRNA abundance of the muscarinic acetylcholine receptors *CHRM1* (*P* < 0.001) and *CHRM5* (*P* < 0.001) and the nicotinic acetylcholine receptor *CHRNA7* (*P* < 0.001) increased linearly as choline supplementation increased; LPS challenge did not affect the response to choline.

### Stage of lactation regulates the response of immune cells to increasing dose of choline

The functional measures of immune cells (phagocytosis, oxidative burst, concentrations of TNF-α, lymphocyte proliferation) were not altered by stage of lactation or its interaction with choline (*P* > 0.10). Nevertheless, lesser (*P* ≤ 0.02) mRNA abundance of *TLR4* and *NFKB1* were observed in neutrophils from cows in early compared with mid-lactation, consistent with the use of neutrophils from cows in early lactation as a model of immunosuppression. Although the abundance of *ELANE* increased with LPS, LPS-challenged neutrophils from cows in early lactation only reached abundance levels similar to that of unchallenged neutrophils from cows in mid-lactation (stage of lactation × LPS, *P* = 0.005, Table 1).

Differential responses to choline by stage of lactation were evident in mRNA abundance. Namely, the abundance of *CHRM5* and *CHRNA7* increased linearly (*P* = 0.01, Table 5) with increasing doses of choline in neutrophils from early- but not from mid-lactation cows. In monocytes, increasing doses of choline increased or tended to increase abundance of *SLC5A7, CHDH, CHAT, CHRM1, CHRM5*, and *CHRNA7* to a greater extent in mid-lactation compared to early lactation (*P* ≤ 0.08, Table 6). Among genes involved in inflammatory response, only neutrophil *CASP3* was impacted by the interaction of choline × stage of lactation (Tables 5, 6). Treatment with CHOL8 suppressed *CASP3* mRNA abundance in early lactation, but enhanced it in mid lactation neutrophils (*P* = 0.005, Table 5).

**Table 5:** Effect of stage of lactation (SL) on the mRNA abundance (2^−ΔCt^ × 10^−3^ AU) of genes involved in choline metabolism and inflammatory response in neutrophils from lactating dairy cows supplemented with choline *in vitro*^a^^,^^b^.

	**Early-lactation**			**Mid-lactation**			**SEM^c^**	***P*****-value**	
	**CHOL3**	**CHOL8**	**CHOL13**	**CHOL3**	**CHOL8**	**CHOL13**		**Linear × SL**	**Quadratic × SL**
**CHOLINE METABOLISM**									
*SLC5A7*	0.79	1.12	0.93	0.69	1.32	0.75	0.40	0.77	0.26
*CHDH*	0.80	1.24	1.28	0.78	1.08	0.81	0.35	0.15	0.69
*CHKA*	236	256	260	220	246	238	38	0.72	0.49
*ACHE*	1.93	2.19	2.21	2.46	2.83	2.73	0.61	0.87	0.84
*CHRM5*	0.51	0.78	0.74	0.84	0.97	0.66	0.24	0.03	0.94
*CHRNA7*	0.63	1.76	1.27	0.90	1.32	0.62	0.56	0.01	0.75
**INFLAMMATORY RESPONSE**									
*TLR4*	970	970	911	2011	2104	1945	152	0.52	0.64
*NFKB1*	929	912	933	1253	1284	1277	101	0.73	0.33
*TNFA*	411	357	378	421	425	446	95	0.21	0.43
*ELANE*	69.4	64.6	67.1	95.9	96.9	95.7	27.8	0.66	0.28
*H2A*	167	167	199	240	236	240	33	0.61	0.25
*CASP3*	39.1	22.8	33.6	34.8	41.0	34.2	7.5	0.59	0.005
*CASP7*	81.0	77.1	85.9	105.7	108.7	96.7	18.4	0.29	0.22

a *Mean values per treatment correspond to cells from cows in 16 cows (8 in early and 8 in mid-lactation) that were or not challenged with LPS (1 μg/mL)*.

b *Cells were cultured with either 3.2 (CHOL3), 8.2 (CHOL8), or 13.2 (CHOL13) μM choline*.

c *Pooled standard error of the mean*.

**Table 6:** Effect of stage of lactation (SL) on the mRNA abundance (2^−ΔCt^ × 10^−3^ AU) of genes involved in choline metabolism and inflammatory response in monocytes from lactating dairy cows supplemented with choline *in vitro*^a^^,^^b^.

	**Early-lactation**			**Mid-lactation**			**SEM^c^**	***P*****-value**	
	**CHOL3**	**CHOL8**	**CHOL13**	**CHOL3**	**CHOL8**	**CHOL13**		**Linear × SL**	**Quadratic × SL**
**CHOLINE METABOLISM**									
*SLC5A7*	0.50	0.55	0.74	0.33	0.63	0.96	0.13	0.001	0.36
*CHDH*	0.59	0.62	0.77	0.47	0.74	0.89	0.11	0.08	0.25
*CHKA*	18.1	19.8	17.3	22.2	21.7	18.3	2.6	0.11	0.65
*ACHE*	1.80	1.70	1.84	2.07	2.11	2.04	0.42	0.65	0.20
*CHAT*	0.19	0.23	0.35	0.10	0.27	0.38	0.08	0.06	0.19
*CHRM1*	0.10	0.11	0.15	0.05	0.11	0.18	0.03	<0.01	0.28
*CHRM5*	0.19	0.21	0.28	0.12	0.26	0.32	0.05	0.03	0.15
*CHRNA7*	0.30	0.36	0.52	0.15	0.38	0.75	0.12	0.01	0.51
**INFLAMMATORY RESPONSE**									
*TLR4*	79.9	82.9	64.7	74.1	64.8	55.2	22.1	0.58	0.24
*NFKB1*	320	310	270	323	309	262	45	0.75	0.95
*TNFA*	150	161	159	135	165	135	30	0.75	0.32
*H2A*	170	184	163	167	180	165	17	0.76	0.80
*CASP3*	6.77	7.11	6.22	6.44	6.47	6.10	1.02	0.78	0.56
*CASP7*	6.55	7.14	6.56	8.61	8.59	8.06	1.04	0.55	0.59

a *Mean values per treatment correspond to cells from 16 cows (8 in early and 8 in mid-lactation) that were or not challenged with LPS (1 μg/mL)*.

b *Cells were cultured with either 3.2 (CHOL3), 8.2 (CHOL8), or 13.2 (CHOL13) μM choline*.

c *Pooled standard error of the mean*.

## Discussion

The current study used isolated immune cells to assess impacts of choline on functional and transcriptional responses *in vitro* to avoid the potential confounding effects of metabolic shifts induced by choline. In general, we found evidence of anti-inflammatory responses to additional choline in innate immune cells, whereas the proliferation rate of lymphocytes was increased by increasing doses of choline. Choline can be metabolized to several other products, including betaine, phosphatidylcholine, and acetylcholine, each with critical biological roles. We measured the mRNA abundance of enzymes involved in the synthesis of these products or their corresponding receptors and transporters, and found that they were most abundant in cells exposed to greater choline concentrations, generally in a quadratic response for neutrophils and a linear response in monocytes.

Two recent papers from the same study evaluated the effect of dietary choline supplementation on dairy cows during the transition from gestation to lactation and failed to observe significant effects of choline on the phagocytic and killing capacity of blood neutrophils (18, 32). On the other hand, the killing capacity of monocytes was greater with increasing doses of choline (18) or with a combination of choline and methionine (32). In contrast, the current study found functional and molecular evidence to imply that increasing doses of choline attenuate the inflammatory response of neutrophils and monocytes. Therefore, the responses observed in the aforementioned *in vivo* study may be a result of indirect effects of one or more products of choline metabolism, coupled with a high variation in the metabolic status of cows in early lactation.

It is important to note that concentrations of choline employed in this experiment were marginally supraphysiological. Although abomasal infusion of choline in cows resulted in plasma choline ion concentrations in line with our CHOL13 treatment (22), other studies suppling choline in the form of RPC generally have had little impact on plasma choline ion concentrations (22, 33). These findings suggest that reported impacts of choline supplementation on animal health are likely mediated through downstream products, whether those effects are primarily on immune cells or not. Still, our treatments are relevant because (a) the infusion results of De Veth et al. (22) demonstrate that these changes in choline ion concentrations are achievable, even if not yet via dietary means, and (b) supplying choline to isolated cells still allows us to address the general question of whether choline or its metabolic products directly influence immune cells. That being said, our findings clearly need to be followed up with more investigation into what metabolic products are likely to be critical to the responses we observed.

The finding that increasing choline concentration enhances the proliferative response of Con A-stimulated lymphocytes appears to conflict with the attenuated inflammatory response observed in choline-supplemented monocytes and neutrophils. However, given the variety of choline products and their potential role in immune cell responses, the current findings suggest that choline can either potentiate or attenuate responses of immune cells, and thus may have an immunomodulatory role. One reason for these divergent responses may be the diversity of choline metabolites which can be produced and trigger their own signaling effects.

In rodents, dietary betaine inhibited ROS generation and reduced the mRNA abundance of cytokines and enzymes involved in pro-inflammatory responses in the tumorigenic colonic mucosa (19). In contrast, mucosa from chicks infected with *Eimeria acervulina* and fed betaine had a greater number of lymphocytes in infected mucosa as well as greater phagocytic and nitric oxide-production capacities of blood monocytes and heterophils [equivalent to neutrophils (34)]. This differential response appears to be mediated by the type of inflammatory/infectious trigger. In chronic inflammation, betaine appears to inhibit inflammation, whereas during an acute infection it promotes an inflammatory response. In the current study, the mRNA abundance of *CHDH*, the enzyme responsible for the synthesis of betaine aldehyde from choline, was increased due to choline supplementation, suggesting that neutrophils and monocytes may be able to synthesize betaine from choline. Although studies evaluating the activity of CHDH and/or the oxidation of choline to betaine in immune cells are scarce, one study reported CHDH activity in normal human monocytes, neutrophils, eosinophils, and basophils, with eosinophils, monocytes, and neutrophils exhibiting greatest activity (35). More recently, the abundance of *CHDH* was reported for human umbilical cord blood-derived monocytes (36). Among livestock, circulating PMN from 3-weeks-old calves did not have detectable mRNA for genes involved in the synthesis of betaine [*CHDH* and *BHMT* (37)]. It is noteworthy that pre-ruminants compared with adult ruminants have greater hepatic activity of betaine-homocysteine methyltransferase (38), perhaps explaining why expression of this enzyme (CHDH) in immune cells is unnecessary in calves.

In the current study, the mRNA abundance of choline kinase α, the enzyme catalyzing the first step in phosphatidylcholine biosynthesis, was increased with choline supplementation in neutrophils but was decreased when the highest dose of choline was supplemented to monocytes. Under the conditions of the current study, with M-199 media that contained free choline only, an increased abundance of *CHKA* may indicate that synthesis of phosphocholine rather than its breakdown was preferred. A comparison of the abundance of *CHDH, CHKA, ACHE*, and *CHAT* indicate that *CHKA* was the most abundant gene (*P* < 0.001) in both neutrophils (abundance > 100 × relative to *CHDH* or *ACHE*) and monocytes (abundance > 5 × relative to *CHDH, ACHE*, or *CHAT*). This identification of the most abundant transcript pool, as a proxy for enzyme abundance, hints at phosphatidylcholine as a potential mediator of the anti-inflammatory effect of increasing doses of choline in bovine neutrophils.

Rats fed phosphatidylcholine and challenged with LPS *in vivo* had an attenuated inflammatory response, including lesser serum concentrations of TNF-α and IL-6 and decreased neutrophil infiltration (39). Similar anti-inflammatory/hyporeactive effects of phosphatidylcholine were observed in neutrophil-dependent acute arthritis in rats (40) and LPS-challenged monocytes from obese human subjects (41). However, intestinal epithelial cells cultured with *C. difficile* and supplemented with phosphatidylcholine had a more robust inflammatory response (20). Furthermore, LPS-challenged macrophages from mice lacking choline cytidyltransferase, a limiting enzyme in phosphatidylcholine synthesis, have reduced capacity to secrete inflammatory cytokines, which is restored after supplementation with phosphatidylcholine (42). Eukaryotic cells can synthesize phosphatidylcholine *de novo*, and immune cells appear to regulate the synthesis and degradation of phosphatidylcholine based on their stimulation state (43–45).

The cholinergic anti-inflammatory pathway was proposed as a mechanism by which the neurotransmitter, acetylcholine, attenuates systemic inflammatory responses via nicotinic receptors (46). Later, the muscarinic acetylcholine receptor was identified as having the opposite effect of nicotinic receptors on the inflammatory response of T cells (47). Acetylcholine and the enzymes involved in its synthesis (*CHAT*) and degradation (*ACHE*), as well as its cholinergic receptors, have been widely measured in lymphocytes (6) and monocytes (7) from rodents. Furthermore, others have also found them to be present in neutrophils from human subjects (8, 48). In the current study, *CHAT*, responsible for the synthesis of acetylcholine, was undetectable in neutrophils, but was detected and increased due to choline supplementation in monocytes. These findings are consistent with a study reporting a 100-fold lesser content of acetylcholine in neutrophils compared with mononuclear cells (lymphocytes and monocytes) from human subjects (8). The mRNA abundance of muscarinic (*CHRM1* and *CHRM5*) and nicotinic (*CHRNA7*) acetylcholine receptors were increased with choline supplementation, though these responses varied with dose and stage of lactation. The mRNA abundance of *CHRNA7* tended to be greater than that of *CHRM5* in neutrophils (*P* = 0.06) and was more abundant than *CHRM5* and *CHRM1* in monocytes (both *P* < 0.001). Greater transcript abundance of the nicotinic vs. muscarinic acetylcholine receptors aligns with the reported anti-inflammatory effects of signaling through this receptor (46, 49). Further studies are necessary to determine whether the anti-inflammatory effect of choline in monocytes is primarily mediated by cholinergic activation of nicotinic receptors and whether phosphatidylcholine synthesis also plays a role. In rats, dietary choline prevented endotoxic shock by blunting Ca^2+^ release and TNF-α production, thus reducing macrophage activation (50). The authors hypothesized that an increase in membrane phosphatidylcholine content may be the mechanism by which dietary choline had an anti-inflammatory effect after an endotoxic shock.

Although not explored in our study, there is evidence of bidirectional crosstalk between cytokine and cholinergic signaling. For example, activation of muscarinic acetylcholine receptors increased IL-2 release by lymphocyes (51), whereas IL-1β suppresses acetylcholine concentrations in the central nervous system (52, 53). This kind of cross-talk, potentially mediated by autocrine interleukin signaling, may underlie the LPS × choline interaction observed for *CHRM5* mRNA abundance in neutrophils.

Recently, Lewis et al. (54) reported that *ex vivo* Con A-challenged splenocytes from suckling rat pups supplemented with phosphatidylcholine, rather than with free choline, increased the rate of proliferation and the production of several cytokines (IL-2, IL-6, and IFN-ɤ). This response occurred with no change in the concentration of choline ion in milk, although phosphatidylcholine concentration increased with supplementation. The authors suggested that the absorption of phosphatidylcholine may be greater than that of free choline, and therefore may be more efficacious for immunomodulation *in vivo*.

The same laboratory found that pups from dams fed a mixture of choline products (glycerophosphocholine, phosphatidylcholine, and free choline) gained more weight and their Con A-challenged splenocytes had greater proportions of helper T cells but produced fewer IFN-ɤ and TNF-α compared with pups from dams fed only free choline (55). These results imply that choline products and quantity fed modulate inflammatory response differently, potentially mediated by the activity of the enzymes involved in their synthesis and/or hydrolysis. In our current *in vitro* study, free choline was provided, which is readily available for cell uptake in culture, with the aim to observe direct signaling effects as well as provide substrate for downstream effects. Given our findings, future studies in cattle should consider the inclusion of choline products alone or in combination to determine whether one or more products of choline metabolism generate responses similar to those observed with free choline. In our study, choline enhanced the proliferation of T cells; unfortunately, due to the design of the study, proliferated lymphocytes were not harvested for downstream analysis of mRNA. Previous studies, summarized by Kawashima and Fujii (56), reported that lymphocytes contain acetylcholine, have acetylcholine-synthesizing activity, and express *CHAT* and different subtypes of cholinergic muscarinic and nicotinic receptors, concluding the existence of an active non-neuronal cholinergic system. Indeed, a study evaluating the role of different T cells stimulants (analogs of Con A) on T cell cholinergic activity found that the mRNA abundance of *CHAT* was increased as well as the synthesis of acetylcholine, which was coupled with upregulation of multiple muscarinic receptors (57). Therefore, this system may be the primary means by which choline enhanced bovine T cell proliferation.

Stage of lactation can impact the inherent competence of immune cells (10), the relative maturity of circulating neutrophils (58), and basal concentrations of choline products in circulation (21). Therefore, we aimed to determine whether immune cells from cows in early- and mid-lactation differ in their responses to increasing doses of choline. Although most of the functional measures of immune cells (phagocytosis, oxidative burst, concentrations of TNF-α, lymphocyte proliferation) were altered by increasing doses of choline, these responses did not interact with stage of lactation. The practical relevance of dietary choline in supporting the health of gestating (59) and lactating (15) dams has been reported in multiple species. In general, these studies have suggested that choline enhances the functionality of the immune system and/or decreases clinical disease incidence. However, *in vivo* treatment has numerous ripple effects on systemic physiology that could indirectly affect immune function of the animal, including altered lipid metabolism and prevention of oxidative stress (60, 61). The current findings clarify our understanding of the impact of choline on immune cells, regardless of whether they are mediated by downstream metabolites or not. It is also worth noting that choline's impacts on immunity may be most apparent during gestation and lactation. These states increase the demand for methyl donors like choline and enhance lipid metabolism (increasing demand for phosphatidylcholine), and total concentrations of choline-derived compounds are substantially decreased in early lactation cows not supplemented with choline (21). As with many nutrients, an evolving understanding of choline's role in immunity should contribute to efforts to define or refine requirements across life stages. A limitation of the current study is that the isolation of immune cells from the whole animal system, although a requirement to investigate the direct impact of choline, also removed interactions with other cells and organs that may regulate choline availability.

**Figure 5:**
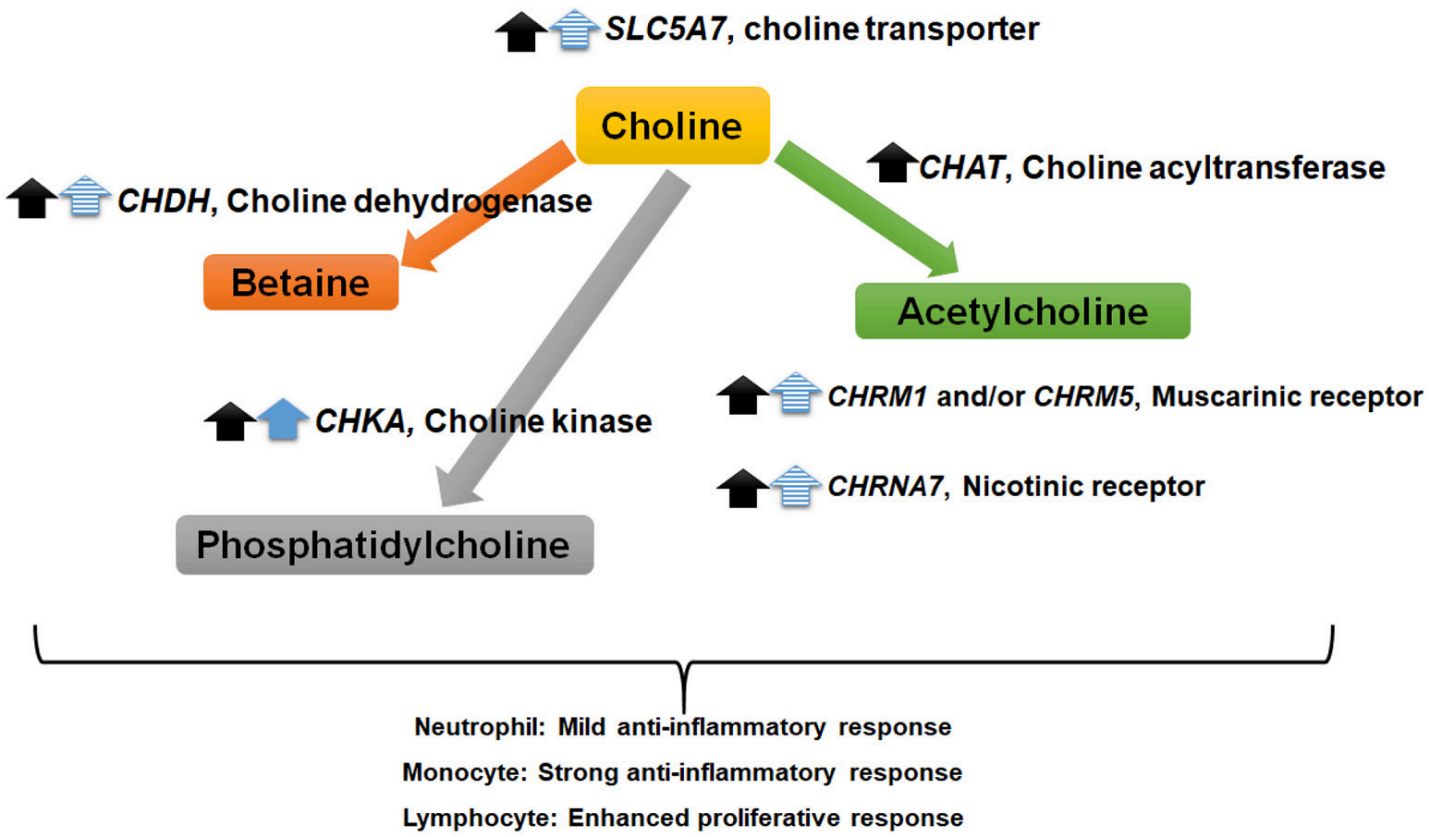
Summary of findings suggesting choline “feed-forward” regulation of choline metabolism and signaling factors in immune cells. Black and blue arrows indicate an increase in mRNA abundance due to increasing doses of choline in monocytes (*P* < 0.01) and neutrophils (*P* < 0.10), respectively. The solid and dashed arrows correspond to a linear and quadratic response to choline, respectively. Monocytes and neutrophils from 18 cows (9 early and 9 mid-lactation cows) were supplemented with choline increasing doses of choline (3.2, 8.2, or 13.2 μM).

In conclusion, we found that the activity of innate and adaptive immune cells from lactating cows is altered by exposure to choline *in vitro*. The detection of mRNA involved in the synthesis of choline products raises the possibility that the observed cell type-dependent inflammatory shifts are regulated by at least one choline product rather than the parent compound itself (Figure 5). Further work is required to determine whether downstream metabolism of choline is required for its effects on specific immune cells.

## Author's note

This article is contribution no. 18-276-J from the Kansas Agricultural Experiment Station.

## Author contributions

MG, BB, and BJB designed the study. MG and LKM performed the experiment and MG analyzed the data. MG and BJB wrote the manuscript. All authors interpreted the data and reviewed the manuscript.

### Conflict of interest statement

The authors declare that the research was conducted in the absence of any commercial or financial relationships that could be construed as a potential conflict of interest.

## Abbreviations

AU: arbitrary units
CHOL3: control treatment, with 3.2 μM choline
CHOL8: treatment with 8.2 μM choline
CHOL13: treatment with 13.2 μM choline
Ct: Threshold cycle
LSM: least square mean
PBMC: Peripheral blood mononuclear cells
PMN: Polymorphonuclear cells
qPCR: quantitative real-time PCR
ROS: Reactive oxygen species
RPC: Rumen-protected choline
SEM: Standard error of the mean.
