# Supplementary material for: Choline Regulates the Function of Bovine Immune Cells and Alters the mRNA Abundance of Enzymes and Receptors Involved in Its Metabolism in vitro
Source: Front Immunol. 2018 Oct 25;9:2448. doi: 10.3389/fimmu.2018.02448 (PMC6211314; doi:10.3389/fimmu.2018.02448)
Supplement: Supplementary file 1 [file Table_1.DOCX]

Supplemental Table 1: Sequence, accession number, and efficiency (E) of the dilution curve^1^

| **Symbol** | **Sequence (5’ to 3’)** | **Accession No.** | **Dilution curve** | | |
| --- | --- | --- | --- | --- | --- |
|  |  |  | Slope | R^2^ | E |
| *RPS9* | F: GAACAAACGTGAGGTCTGGAGG | NM_001101152.2 | -3.28 | 1.00 | 1.02 |
|  | R: TTACCTTCGAACAGACGCCG |  |  |  |  |
| *RPS15* | F: GGCGGAAGTGGAACAGAAGA | NM_001024541.2 | -3.60 | 1.00 | 0.90 |
|  | R: GTAGCTGGTCGAGGTCTACG |  |  |  |  |
| *SLC5A7* | F: TGGACAAGACGATTCTGGTTAG | NM_001046116.2 | -3.41 | 1.00 | 0.97 |
|  | R: GAAGGTCGAGCTGAGAGTTATG |  |  |  |  |
| *CHDH* | F: CGGCTGGCTAATTCTCATTCT | NM_001205564.1 | -3.20 | 1.00 | 1.05 |
|  | R: GGACGAGGATCATAGCTCTACT |  |  |  |  |
| *CHKA* | F: CTTACCTCGTTTACGACCTCAC | XM_015470088.1 | -3.59 | 1.00 | 0.90 |
|  | R: TAGATGAGAGGGTCTGAGATACTG |  |  |  |  |
| *ACHE* | F: CAGTACGTGAGCCTGAATCTG | NM_001076220.1 | -3.34 | 1.00 | 0.99 |
|  | R: CTGAGTAGTTTGGGCAGGAAG |  |  |  |  |
| *CHAT* | F: GCTGGACTGGTAGGAAGAATG | XM_015469891.1 | -3.14 | 1.00 | 1.08 |
|  | R: CAGAGCCAGGCTAGTCTTATTT |  |  |  |  |
| *CHRM1* | F: GAGCTCAAGACGGTCAACAA | NM_001244609.1 | -3.22 | 0.96 | 1.04 |
|  | R: TGAGCAGATACGTGGTGTAAAG |  |  |  |  |
| *CHRM5* | F: ATCCTCTACTGCCGCATCTA | NM_001244611.1 | -3.12 | 1.00 | 1.09 |
|  | R: GTGGGCTGACTTTCTCTTCTC |  |  |  |  |
| *CHRNA7* | F: GAAGTTTGGGTCCTGGTCTTAT | NM_174515.2 | -3.59 | 1.00 | 0.90 |
|  | R: ACAAGGTCCCATTCTCCATTT |  |  |  |  |
| *TLR4* | F: CATGGGCTTAGAGCAACTAGAA | NM_174198.6 | -3.37 | 1.00 | 0.98 |
|  | R: GCGGAGGTTTCTGAGTGATAG |  |  |  |  |
| *NFKB1* | F: ACCTCCTTCCGCAAACTCAG | NM_001076409.1 | -3.44 | 1.00 | 0.95 |
|  | R: ATAGGTCCTTCCTGCCCGTA |  |  |  |  |
| *TNFA* | F: AAGTAACAAGCCGGTAGCCCA | NM_173966.3 | -3.62 | 0.99 | 0.89 |
|  | R: CTTCCAGCTTCACACCGTTG |  |  |  |  |
| *ELANE* | F: GCCAACTGGATCAACTCTATCA | NM_001105653.1 | -3.56 | 1.00 | 0.91 |
|  | R: GACAGGTACAGCCTTTCTAGTG |  |  |  |  |
| *H2A* | F: GGCAGGAAATGCATCGAAAG | NM_174809.2 | -3.36 | 1.00 | 0.98 |
|  | R: GCCTTGATGAGAGAGTCCAAT |  |  |  |  |
| *CASP3* | F: ACAGAACTGGACTGTGGTATTG | NM_001077840.1 | -3.44 | 0.99 | 0.95 |
|  | R: GAAGTCTGCCTCAACTGGTATT |  |  |  |  |
| *CASP7* | F: CATAGTGAGGCAGAGAAGGAAC | XM_604643.7 | -3.50 | 1.00 | 0.93 |
|  | R: ATCGCTGAACACAGGGAATAG |  |  |  |  |

^1^ E: Efficiency was tested with 5 serial dilutions and was calculated as E = (10^(−1/slope)^) −1
