# Supplementary material for: Choline Regulates the Function of Bovine Immune Cells and Alters the mRNA Abundance of Enzymes and Receptors Involved in Its Metabolism in vitro
Source: Front Immunol. 2018 Oct 25;9:2448. doi: 10.3389/fimmu.2018.02448 (PMC6211314; doi:10.3389/fimmu.2018.02448)
Supplement: Supplementary file 2 [file Table_2.DOCX]

Supplemental Table 2: Ct values for genes analyzed in monocytes and neutrophils

| **Gene Symbol** | **Ct values in Monocytes** | | | |  | **Ct values in Neutrophils** | | | |
| --- | --- | --- | --- | --- | --- | --- | --- | --- | --- |
|  | **Min** | **Max** | **Mean** | **Median** |  | **Min** | **Max** | **Mean** | **Median** |
| *RPS9* | 16.72 | 19.63 | 17.73 | 17.76 |  | 18.64 | 22.61 | 20.10 | 20.23 |
| *RPS15* | 16.95 | 19.75 | 17.81 | 17.71 |  | 18.47 | 21.16 | 19.59 | 19.62 |
| *SLC5A7* | 26.55 | 31.16 | 28.50 | 28.46 |  | 30.72 | 33.78 | 29.78 | 29.74 |
| *CHDH* | 26.02 | 29.96 | 28.32 | 28.33 |  | 27.26 | 33.33 | 29.87 | 29.82 |
| *CHKA* | 22.24 | 25.66 | 23.43 | 23.36 |  | 16.62 | 24.74 | 21.89 | 21.78 |
| *ACHE* | 24.71 | 28.77 | 26.81 | 26.85 |  | 25.39 | 32.12 | 28.56 | 28.53 |
| *CHAT* | 27.41 | 34.81 | 29.85 | 29.59 |  | Undetectable | | | |
| *CHRM1* | 30.94 | 34.13 | 30.94 | 30.91 |  | Undetectable | | | |
| *CHRM5* | 28.02 | 33.04 | 29.93 | 29.85 |  | 27.39 | 32.65 | 30.10 | 29.99 |
| *CHRNA7* | 26.33 | 33.27 | 29.18 | 29.18 |  | 26.85 | 34.01 | 29.62 | 29.57 |
| *TLR4* | 19.41 | 24.39 | 21.59 | 21.42 |  | 17.98 | 20.64 | 19.39 | 19.53 |
| *NFKB1* | 18.18 | 21.72 | 19.49 | 19.48 |  | 18.69 | 20.75 | 19.77 | 19.74 |
| *TNFA* | 16.87 | 23.37 | 20.50 | 20.74 |  | 17.20 | 24.56 | 21.19 | 21.18 |
| *ELANE* | Not analyzed | | | |  | 22.11 | 26.29 | 23.50 | 23.29 |
| *H2A* | 19.19 | 21.60 | 20.31 | 20.38 |  | 21.46 | 22.53 | 22.09 | 22.17 |
| *CASP3* | 23.73 | 26.45 | 25.01 | 24.94 |  | 23.84 | 27.69 | 24.77 | 24.45 |
| *CASP7* | 23.05 | 26.60 | 24.82 | 24.78 |  | 22.10 | 24.89 | 23.31 | 23.18 |
